# MepmiRDB: a medicinal plant microRNA database

**DOI:** 10.1093/database/baz070

**Published:** 2019-06-24

**Authors:** Dongliang Yu, Jiangjie Lu, Weishan Shao, Xiaoxia Ma, Tian Xie, Hidetaka Ito, Tingzhang Wang, Min Xu, Huizhong Wang, Yijun Meng

**Affiliations:** 1 College of Life and Environmental Sciences, Hangzhou Normal University, Hangzhou, 310036, China; 2 Zhejiang Provincial Key Laboratory for Genetic Improvement and Quality Control of Medicinal Plants, Hangzhou Normal University, Hangzhou, 310036, China; 3 Department of Pharmacology, Holistic Integrative Pharmacy Institutes, College of Medicine, Hangzhou Normal University, Hangzhou, 311121, China; 4 Key Laboratory of Elemene Class Anti-cancer Chinese Medicine of Zhejiang Province and Engineering Laboratory of Development and Application of Traditional Chinese Medicine from Zhejiang Province, Hangzhou Normal University, Hangzhou, 311121, China; 5 Faculty of Science, Hokkaido University, Sapporo, 060-0810, Japan; 6 Key Laboratory of Microbiological Technology and Bioinformatics Research in Zhejiang Province, Hangzhou, 310012, China

## Abstract

MicroRNAs (miRNAs) have been recognized as a key regulator in plant development and metabolism. Recent reports showed that the miRNAs of medicinal plants not only act as a critical modulator in secondary metabolism but also had a great potential of performing cross-kingdom gene regulation. Although several plant miRNA repositories have been publicly available, no miRNA database specific for medicinal plants has been reported to date. Here, we report the first version of MepmiRDB (medicinal plant microRNA database), which is freely accessible at http://mepmirdb.cn/mepmirdb/index.html. This database accommodates thousands of miRNA candidates belonging to 29 medicinal plant species. The miRNA information on sequences, expression patterns and regulatory networks has been included in the functional modules of the database. Specifically, the ‘Sequence’ module provides the sequences of the mature miRNAs and their precursors, and the structure information of the precursors. Moreover, the processing and small RNA accumulation signals on the miRNA precursors are also included in the ‘Sequence’ module. The organ/growth condition-specific expression information of the mature miRNAs has been stored in the ‘Expression’ module. The ‘Interaction’ module offers the information of the degradome-validated miRNA—target pairs of eight plant species. The ‘Search’ module enables users to search for the miRNAs by plant species and miRNA families, or by sequences. All data in this database are available for download. Taken together, the functional modules of MepmiRDB ensure its importance and timeliness for mechanistic and functional studies on the medicinal plant miRNAs.

## Introduction

MicroRNAs (miRNAs) play critical roles in diverse biological processes of plants, such as growth and development, stress response and metabolism (1). Currently, there are several databases accommodating information of plant miRNAs, such as miRBase (2), PMRD (3), miRNEST (4), miREX (5), PmiRKB (6) and PNRD (7). However, to date, no database has been established specifically for the miRNAs of medicinal plants. Here, we report the first version of MepmiRDB (medicinal plant microRNA database) including 29 medicinal plant species, most of which are listed in the pharmacopoeia of the People’s Republic of China (8).

Increasing pieces of evidence point to the pivotal role of plant miRNAs in modulating the biosynthesis and accumulation of secondary metabolites (9, 10). It is particularly important for the medicinal plants since a dominant portion of the bioactive ingredients are produced through the secondary metabolic pathways (11–14). On the other hand, miRNAs have become a charming candidate for therapeutic management of cancer and other intractable diseases (15). Notably, several reports indicated the ability of plant miRNAs in cross-kingdom communication (16–19), and some studies showed the high stability of certain ingested miRNAs during circulation in human or animal bloodstream (20–22). These lines of evidence point to the possibility that the exogenous, plant-derived miRNAs could perform target regulation in human body. In this regard, the miRNAs emerged as the bioactive components of medicinal plants (23–25). Taken together, the above-mentioned points formed the driving force for design and construction of MepmiRDB, which has been freely available online (http://mepmirdb.cn/mepmirdb/index.html).

**Table 1:** The number of microRNAs reported by the current version of MepmiRDB.

**Organism**	**miR_total**	**miR_miRBase**	**miR_miRDP**	**miR_(miRBase ∩ miRDP)**	**Pri-miR_miRDP**	**Pre-miR_miRDP**
***Actinidia chinensis* (ach)**	**141**	**141**	**0**	**0**	**0**	**0**
***Ammopiptanthus mongolicus* (amo)**	**456**	**332**	**151**	**27**	**148**	**76**
***Aquilaria sinensis* (asi)**	**235**	**202**	**44**	**11**	**23**	**22**
***Catharanthus roseus* (cro)**	**217**	**166**	**68**	**17**	**38**	**37**
***Citrus sinesis* (csi)**	**948**	**470**	**530**	**52**	**641**	**273**
***Carthamus tinctorius* (cti)**	**287**	**250**	**42**	**5**	**22**	**21**
***Dimocarpus longan* (dlo)**	**195**	**154**	**55**	**14**	**30**	**30**
***Dendranthema morifolium* (dmo)**	**497**	**199**	**312**	**14**	**317**	**168**
***Dendrobium officinale* (dof)**	**669**	**283**	**408**	**22**	**370**	**225**
***Eucommia ulmoides* (eul)**	**313**	**313**	**0**	**0**	**0**	**0**
***Ginkgo biloba* (gbi)**	**438**	**298**	**150**	**10**	**106**	**77**
***Juglans regia* (jre)**	**329**	**224**	**148**	**43**	**176**	**88**
***Litchi chinensis* (lch)**	**298**	**219**	**85**	**6**	**84**	**43**
***Lonicera japonica* (lja)**	**315**	**161**	**171**	**17**	**140**	**117**
***Lycium chinense* (lyc)**	**298**	**245**	**67**	**14**	**41**	**35**
***Nelumbo nucifera* (nnu)**	**627**	**453**	**207**	**33**	**198**	**107**
***Oenanthe javanica* (oja)**	**25**	**25**	**0**	**0**	**0**	**0**
***Panax ginseng* (pgi)**	**249**	**117**	**135**	**3**	**167**	**77**
***Punicai granatum* (pgr)**	**261**	**258**	**4**	**1**	**2**	**2**
***Paeonia lactiflora* Dafugui (plaD)**	**192**	**192**	**0**	**0**	**0**	**0**
***Paeonia lactiflora* Hongyanzhenghui (plaH)**	**154**	**154**	**0**	**0**	**0**	**0**
***Paeonia lactiflora* Zifengyu (plaZ)**	**163**	**163**	**0**	**0**	**0**	**0**
***Panax notoginseng* (pno)**	**339**	**281**	**66**	**8**	**45**	**34**
***Papaver somniferum* (pso)**	**164**	**138**	**26**	**0**	**14**	**13**
***Ricinus communis* (rco)**	**178**	**159**	**34**	**15**	**29**	**18**
***Raphanus sativus* (rsa)**	**124**	**120**	**4**	**0**	**2**	**2**
***Salvia miltiorrhiza* (smi)**	**345**	**220**	**143**	**18**	**122**	**75**
***Taxus mairei* (tma)**	**180**	**132**	**49**	**1**	**25**	**25**
***Taxus x media* (tme)**	**581**	**315**	**281**	**15**	**257**	**145**

### Database construction and contents

**Figure 1:**
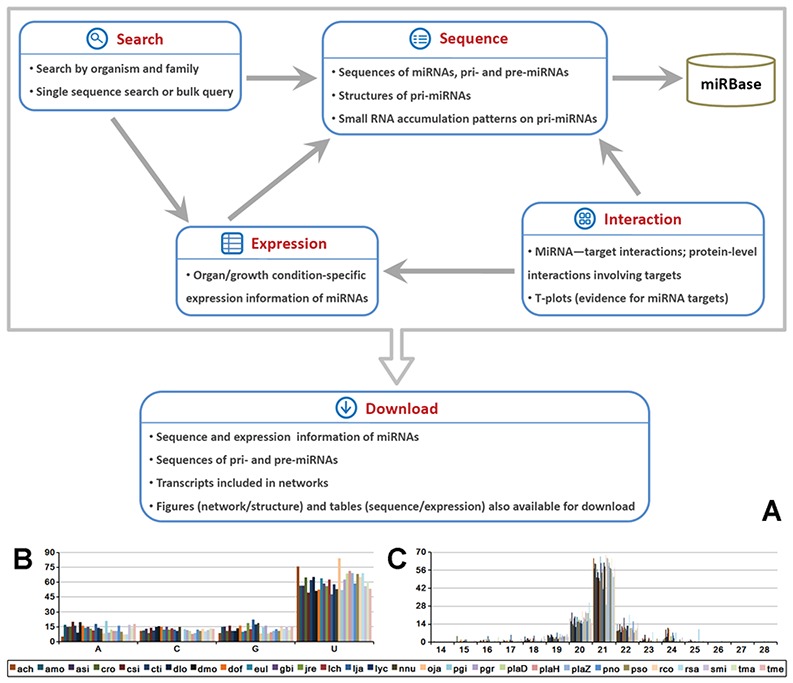
Brief summary of MepmiRDB (medicinal plant microRNA database). (A) Functional flow chart of MepmiRDB. The database provides four major functional modules, ‘Sequence’, ‘Expression’, ‘Interaction’ and ‘Search’. All of the sequence and expression data could be retrieved from ‘Download’. (B) 5′ first nucleotide composition of the miRNAs deposited in MepmiRDB. (C) Sequence length distribution of the miRNAs deposited in MepmiRDB. For (B) and (C), the 29 plant species are indicated by different colors, as shown on the bottom.

The database structure has been illustrated in Figure S1 and S2. Five major functional modules, including ‘Sequence’, ‘Expression’, ‘Interaction’, ‘Search’ and ‘Download’, are available in MepmiRDB (Figure 1A) and are described below.

### The ‘Sequence’ module

**Figure 2:**
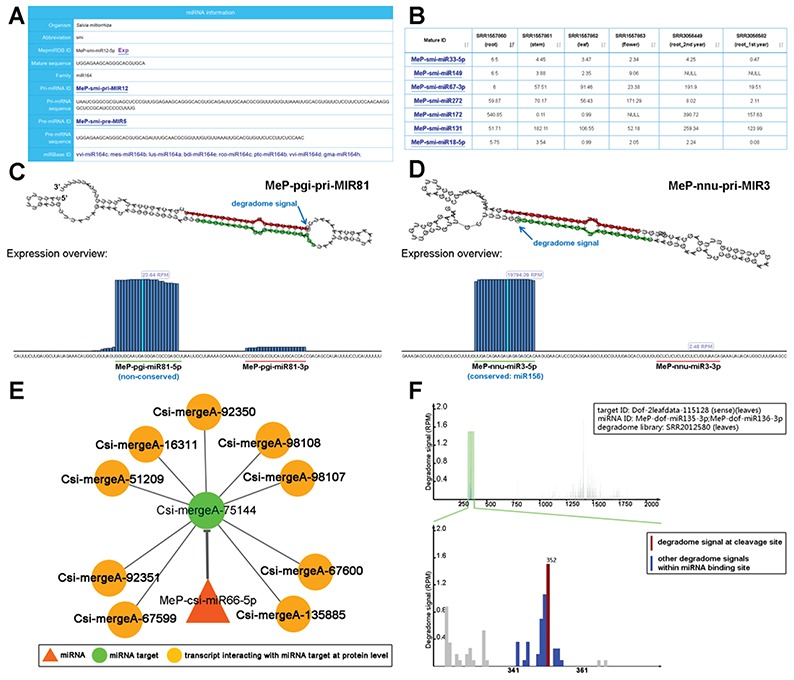
Sample results of different functional modules. (A) Through the ‘Sequence’ module, the sequence information of the mature miRNA (miRNAs; MeP-smi-miR12-5p for example here) of *S. miltiorrhiza* (smi), along with its precursors (MeP-smi-pri-MIR12 and MeP-smi-pre-MIR5), could be obtained. Based on sequence conservation, the miRNA has been assigned to the miR164 family. The ‘miRBase ID’ provides external links of miRNA homologs. (B) Through the ‘Expression’ module, miRNA expression levels in different organs (root, stem, leaf and flower) of *S. miltiorrhiza* could be browsed as a table, which is downloadable. The ‘Mature ID’ provides links for visiting the ‘Sequence’ module. (C) Processing and small RNA (sRNA) expression signals on MeP-pgi-pri-MIR81. Two regions encoding mature miRNAs (MeP-pgi-miR81-5p and MeP-pgi-miR81-3p) on the predicted secondary structure were marked by green and red lines respectively. The processing signal detected by degradome-seq data analysis was denoted by a blue arrow. The expression level of the sRNAs on the pri-miRNA was measured by RPM (reads per million). (D) Processing and sRNA expression signals on MeP-nnu-pri-MIR3. Two regions encoding mature miRNAs (MeP-nnu-miR3-5p and MeP-nnu-miR3-3p) on the predicted secondary structure were marked by green and red lines respectively. The processing signal detected by degradome-seq data analysis was denoted by a blue arrow. The expression level of the sRNAs on the pri-miRNA was measured by RPM. (E) Example of regulatory network constituted by a miRNA (MeP-csi-miR66-5p), its targets and the interacting transcripts. (F) T-plot (target plot) showing the cleavage signals of the miRNA–target regulatory pair. The upper panel provides a global view of the degradome signals (the leaf degradome library) on the full-length target transcript (Dof-2leafdata-115 128). The light green shadow indicates the binding site of the regulatory miRNAs (MeP-dof-miR135-3p and MeP-dof-miR136-3p). The lower panel provides the local view of the degradome signals (indicated by blue bars) within the miRNA binding region. The cleavage signal was highlighted by red color. The positions of the miRNA binding region and the cleavage signal were also shown on the lower panel. The degradome signal intensity was measured by RPM (reads per million).

Sequence information of thousands of the miRNAs belonging to 29 plant species was included in this module. All of the miRNAs were predicted by using the recently published workflow PmiRDiscVali (26). These miRNAs were generally divided into conserved and non-conserved ones. By comparing to all of the plant miRNAs registered in miRBase (release 21), the sRNAs with identical sequences were extracted from the sRNA-seq (small RNA sequencing) data sets, and were regarded as the conserved miRNAs existed in specific medicinal plants (Figure 2A for example). Besides, 86 RNA-seq datasets of 23 plant species were employed for transcriptome assembly by Trinity (trinityrnaseq_r20140717, default parameters) (27). The assembled transcripts were served as the basic data for miRNA precursor discovery by using miRDeep-P (v1.3, default parameters) (28). We noticed that a large portion of the miRDeep-P predictions belonged to the non-conserved miRNAs (Table 1), suggesting that these miRNA candidates might be evolutionarily young or species-specific.

An overview of the sequence characteristics shows that around 50% of the miRNA candidates start with 5’ U and are 21 nt in length (Figure 1B and C), which is consistent with the known sequence features of plant miRNAs (1).

Degradome-seq data is useful for tracking the processing signals of miRNA precursors (29, 30). For eight plant species with degradome-seq data, the ‘Sequence’ module provides the detectable processing signals on the secondary structures of the pri-miRNAs (Figure 2C and D). Additionally, sRNA expression levels on the miRNA precursors were achieved by mapping the sRNA-seq data onto the pri-miRNAs. In many cases, the highly enriched accumulation regions of the sRNAs correlated well with the mature miRNA-coding regions, indicating the high reliability of the pri-miRNA candidates (Figure 2C and D).

### The ‘Expression’ module

The module provides the organ/growth condition-specific expression information of the mature miRNA candidates (Figure 2B).

The ‘Interaction’ module

For the eight species with degradome-seq data, miRNA target prediction was performed by using the psRNATarget algorithm (2017 Update, default parameters) (31), treating the assembled transcripts and the mature miRNA candidates as the input data. Then, the predicted miRNA–target pairs were subject to degradome-seq data-based validation according to the previously reported method (32). As a result, 380 pairs involving 119 miRNAs and 47 target transcripts in *Ammopiptanthus mongolicus*, 2341 pairs involving 192 miRNAs and 208 targets in *Citrus sinesis*, 13 023 pairs involving 389 miRNAs and 4506 targets in *Dendrobium officinale*, 6933 pairs involving 216 miRNAs and 2968 targets in *Nelumbo nucifera*, 9660 pairs involving 143 miRNAs and 2009 targets in *Panax ginseng*, 1560 pairs involving 130 miRNAs and 864 targets in *Salvia miltiorrhiza*, 149 pairs involving 72 miRNAs and 55 targets in *Taxus mairei* and 262 pairs involving 22 miRNAs and 98 targets in *Taxus* × *media* were identified. For each pair, t-plot (target plot) showing target cleavage signal(s) and visual network showing regulatory relationship between the miRNA and its target are available for users (Figure 2E and F). Besides, the transcripts interacting with the miRNA targets at the protein level were retrieved from the STRING database (33) by using BLASTX (34) (v2.2.26, *E*-value <1e-3, query coverage >50%, identity >35%). As a result, the protein–protein interactions were also visible in the regulatory networks (Figure 2E).

### The ‘Search’ module

The module enables users to search for the miRNAs by plant species and miRNA families, or by sequences. For sequence search, it enables users to perform either ‘exact’ or ‘fuzzy’ search.

### Other functional modules

The ‘Statistics’ module provides users with statistical information in both table and figure formats. Besides, all of the sequence and expression information reported in MepmiRDB has been made freely available in the ‘Download’ module. A help document is available in the ‘Help’ module for the first-time visitors.

## Conclusion

All of the modules provided by MepmiRDB ensure its importance and timeliness for mechanistic and functional studies on the medicinal plant miRNAs. It is foreseeable that MepmiRDB will attract wide attention from medical and pharmaceutical researchers, and plant biologists.

## Availability

MepmiRDB database is freely available at http://mepmirdb.cn/mepmirdb/index.html. We recommend the visitors to use the browsers like Mozilla Firefox, Google Chrome and Safari.

## Supplementary data

Supplementary data are available at *Database* Online.

## Supplementary Material

Figure_S1_baz070

Figure_S2_baz070

## Notes

**Database URL**: http://mepmirdb.cn/mepmirdb/index.html
