# Supplementary material for: MepmiRDB: a medicinal plant microRNA database
Source: Database (Oxford). 2019 Jun 24;2019:baz070. doi: 10.1093/database/baz070 (PMC6589547; doi:10.1093/database/baz070)
Supplement: Figure_S1_baz070 [file figure_s1_baz070.pdf]

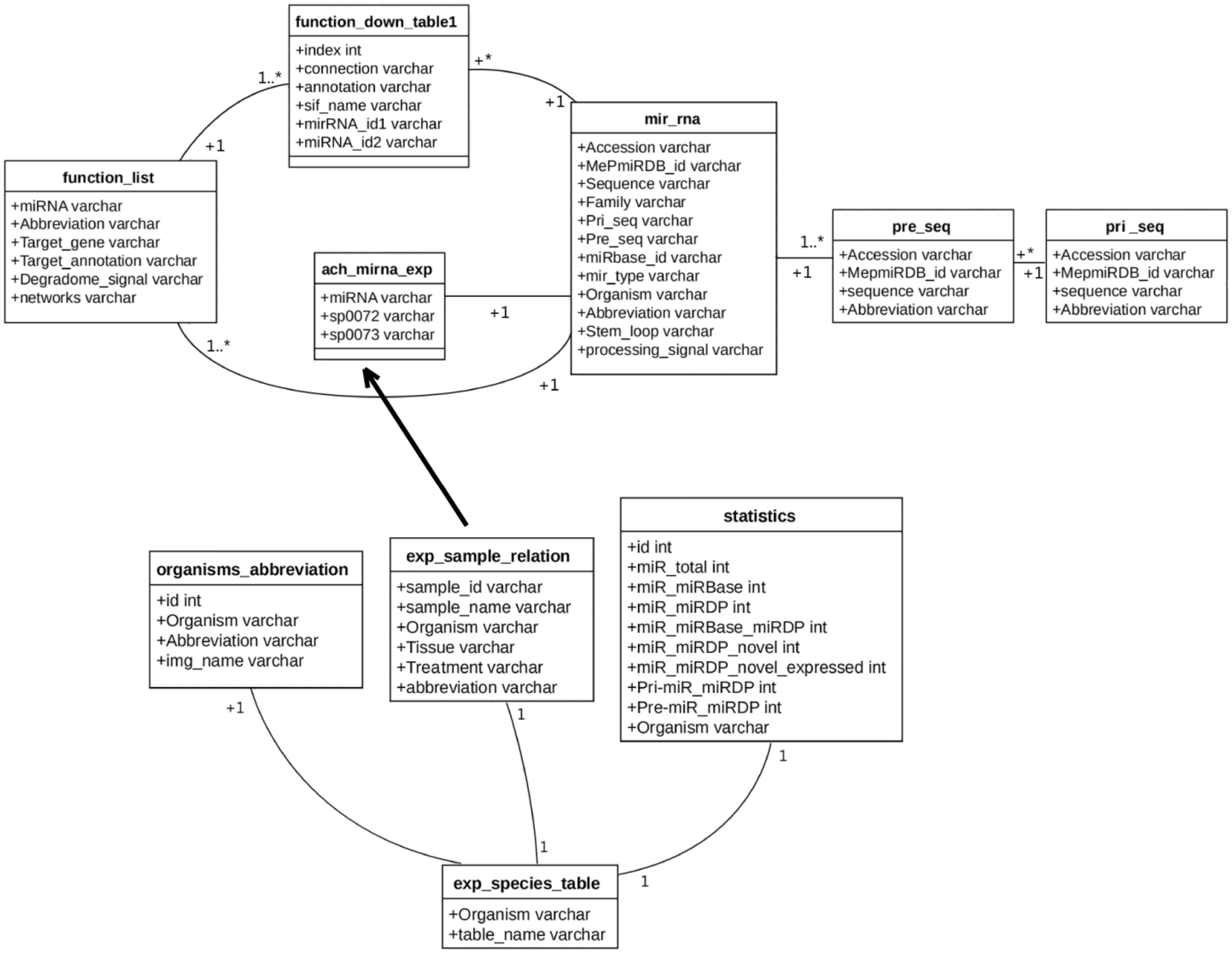

Figure S1 Background structure of MepmiRDB. Sequence information of pri-/pre-/mature miRNAs and the expression of mature miRNAs in diverse tissues are indexed and stored in MySQL database. Expression profiles are created as individual tables for each organism.
