# Supplementary material for: MepmiRDB: a medicinal plant microRNA database
Source: Database (Oxford). 2019 Jun 24;2019:baz070. doi: 10.1093/database/baz070 (PMC6589547; doi:10.1093/database/baz070)
Supplement: Figure_S2_baz070 [file figure_s2_baz070.pdf]

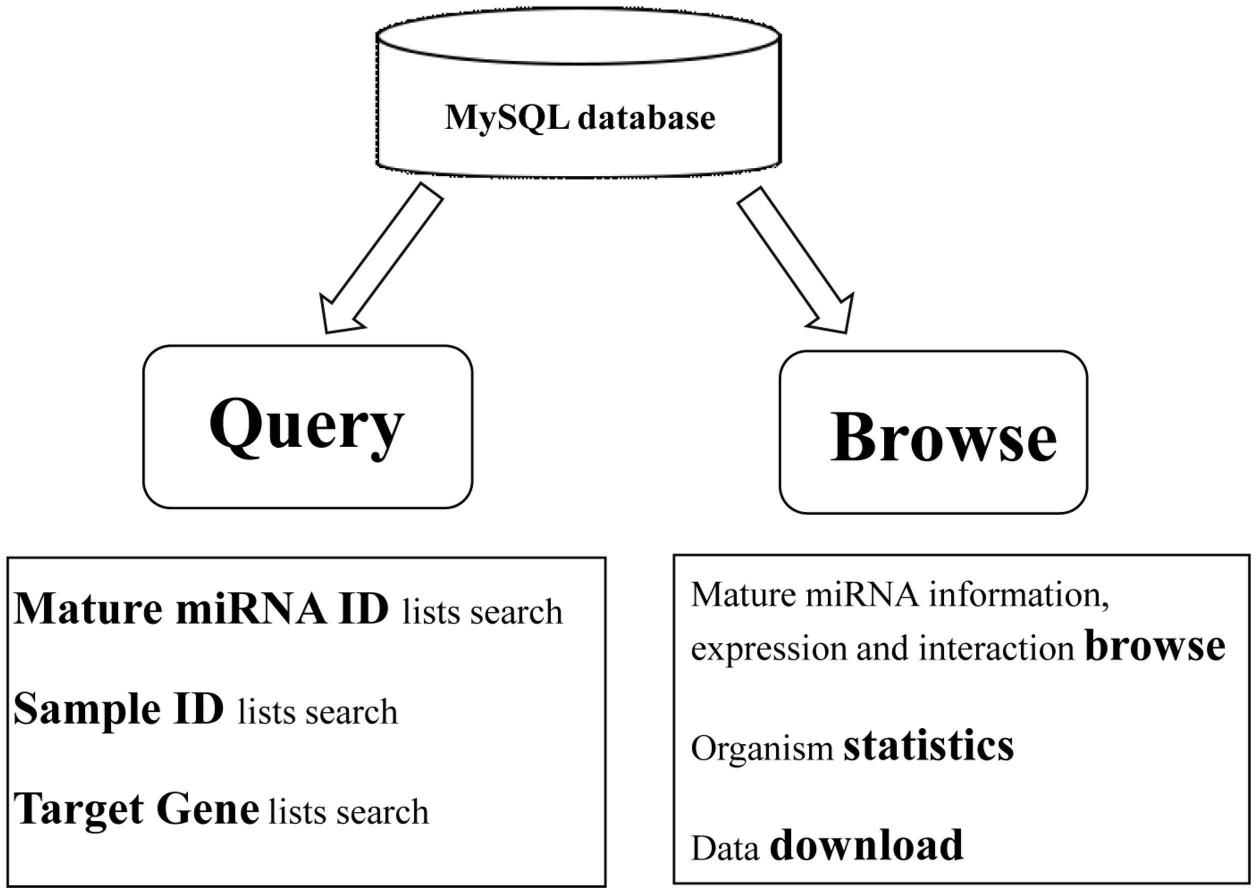

Figure S2 Functional architecture of the MepmiRDB. MepmiRDB is an open-access database that provides the web service for browsing and querying the information of the predicted miRNAs, especially on their sequences, structures, expression profiles and interaction networks.
